# Development of a rapid and sensitive RPA-CRISPR/Cas12a-based assay for the detection of *Brucella melitensis*

**DOI:** 10.1128/spectrum.00998-25

**Published:** 2025-08-27

**Authors:** Yu Shen, Chenchen Yi, Heng Wang, Yi Tang, Jun Li

**Affiliations:** 1 Hangzhou Center for Disease Control and Prevention (Hangzhou Health Supervision Institution) 117870, Hangzhou, China; 2 Zhejiang Key Laboratory of Multi-Omics in Infection and Immunity, Hangzhou, China; 3 Center of Reproductive Medicine, Sir Run Shaw Hospital, Zhejiang University School of Medicine 26441, Hangzhou, China; MultiCare Health System, Tacoma, Washington, USA

**Keywords:** *Brucella*, RPA, CRISPR/Cas12a, diagnosis

## Abstract

Brucellosis, a zoonotic disease caused by *Brucella* species, presents significant public health challenges due to its complex diagnosis and the limited availability of rapid detection methods. To address these challenges, we developed a novel detection method that integrates recombinase polymerase amplification (RPA) with the CRISPR/Cas12a system, enabling dual readout through fluorescence (FL) and lateral flow strip (LFS) detection. The RPA-CRISPR/Cas12a-FL assay demonstrated an impressive detection limit of 1 copy/μL, which is 10 times more sensitive than quantitative polymerase chain reaction, while the RPA-CRISPR/Cas12a-LFS method achieved a detection limit of 10 copies/μL, comparable to nested PCR. Specificity testing confirmed the robustness of the assay, as it produced strong signals exclusively for *Brucella* without cross-reactivity with other bacterial species. Clinical validation using serum samples from 24 confirmed brucellosis patients and six healthy controls demonstrated a 100% concordance with serological results, underscoring the reliability of this method for clinical applications. This assay provides a rapid, sensitive, and specific tool for *Brucella* detection, suitable for both laboratory and field settings, and holds significant potential for enhancing the diagnosis and control of brucellosis.

**IMPORTANCE:**

Brucellosis is a significant zoonotic disease, and rapid and accurate diagnosis is crucial for its treatment and control. To address this need, we developed a novel detection method that combines recombinant enzyme polymerase amplification with a CRISPR/Cas12a system, achieving dual readout through fluorescence and lateral flow strips. The test demonstrates excellent sensitivity and specificity, with clinical validation confirming complete concordance with serological results. This approach offers a fast, reliable, and field-deployable solution for brucellosis diagnosis, significantly enhancing disease management and public health outcomes.

## INTRODUCTION

Brucellosis is an easily overlooked zoonotic disease caused by gram-negative, facultative intracellular *Brucella* species (1, 2). It is recognized as one of the most widespread zoonotic bacterial infections globally, with the primary strains associated with human infections being *B. melitensis*, *B. abortus*, *B. suis*, and *B. canis*, among which *B. melitensis* is the most prevalent and pathogenic (1, 3, 4). *Brucella* is usually transmitted to humans through consumption of unpasteurized dairy products or direct contact with infected animals and their secretions (5, 6). Clinically, the disease often manifests with acute flu-like symptoms, including fever, chills, malaise, and fatigue, and may progress to a chronic condition (7). It can also result in various complications, such as undulant fever, arthritis, spondylitis, endocarditis, and chronic fatigue syndrome (8, 9). Due to the non-specific nature of the symptoms, human brucellosis is frequently misdiagnosed as other diseases; thus, accurate diagnosis and treatment are crucial for effective management (10).

Commonly used methods for detecting *Brucella* include bacterial isolation and identification, immunological detection, and nucleic acid detection (11). Of these, the traditional isolation and culture methods, although considered the gold standard, require specialized laboratory conditions and long incubation times to obtain results (12). In addition, the sensitivity of blood cultures may be significantly reduced in cases of chronic diseases and localized infections (12, 13). As a result, immunodiagnostic methods have become more widely used, particularly the Rose Bengal test, the standard tube agglutination test, and the enzyme-linked immunosorbent assay (11). Despite the widespread use of these tests and their rapid results, they have certain limitations. For example, there is a window period for the test, and false-positive results may also occur due to cross-reactivity with other gram-negative bacterial infections, raising concerns about specificity (14, 15).

In recent years, molecular biology methods, such as qualitative and quantitative polymerase chain reaction (qPCR) targeting specific genes, have been widely used for the diagnosis of the disease (16, 17). PCR is a highly sensitive and practical method, capable of amplifying nucleic acids to detect infecting microorganisms in a sample (18). Although PCR-based technology can be used for the diagnosis of brucellosis, its application in resource-poor and high-prevalence areas is limited due to the need for expensive equipment and specialized laboratories. Recombinase polymerase amplification (RPA) is a novel, thermostable nucleic acid amplification technique that provides rapid and efficient amplification of DNA or RNA without the need for complex equipment, making it suitable for field applications and resource-poor environments (19, 20). Previous studies have shown that RPA can effectively detect the *Brucella* bcsp31, bp26, and IS711 genes with excellent sensitivity and specificity (21, 22). However, RPA has some limitations: for example, RPA may produce non-specific amplification, leading to false positives (23).

The CRISPR/Cas system, especially Cas12a, plays a significant role in the field of molecular diagnosis. Cas12a is a bacterial-derived nuclease that binds to a TTTV protospacer adjacent motif (V = A/G/C) upstream of the target DNA and exhibits non-specific cleavage activity, making it suitable for rapid nucleic acid testing (24). The combination of RPA and Cas12a provides higher sensitivity and specificity than Cas12a alone, and RPA can rapidly amplify the target DNA under isothermal conditions, increasing the sensitivity of the Cas12a assay and enabling efficient detection of pathogens at very low levels of pathogenic nucleic acids (25). This method has been widely used for the rapid diagnosis of pathogens such as SARS-CoV-2 (26), *Mycobacterium tuberculosis* (27), and *Plasmodium falciparum* (28), and is suitable for resource-limited areas.

In this study, we designed RPA primers and crRNAs for the *B. melitensis* omp31 gene as a target sequence and developed a corresponding RPA-CRISPR/Cas12a detection system. We developed an RPA-CRISPR/Cas12a assay by combining RPA and CRISPR/Cas12a in a single-tube reaction system. The assay can be performed using either real-time quantitative fluorescence (FL) analysis or lateral flow immunoassay strips, each of which uses a specific ssDNA reporter molecule for signal readout. The specificity of the method was evaluated by testing a variety of other bacteria, and sensitivity was assessed at the level of molecular detection. In addition, the method was applied to serum samples from patients with *Brucella* infections in different hospitals in Hangzhou, Zhejiang Province, China, which validated its potential application in clinical diagnosis. In conclusion, this study provides a novel molecular diagnostic strategy for the rapid and sensitive detection of Brucella and lays the foundation for further optimization of clinical detection methods.

## RESULTS

### Establishment and optimization of RPA-CRISPR/Cas12a one-tube assay

**Fig 1:**
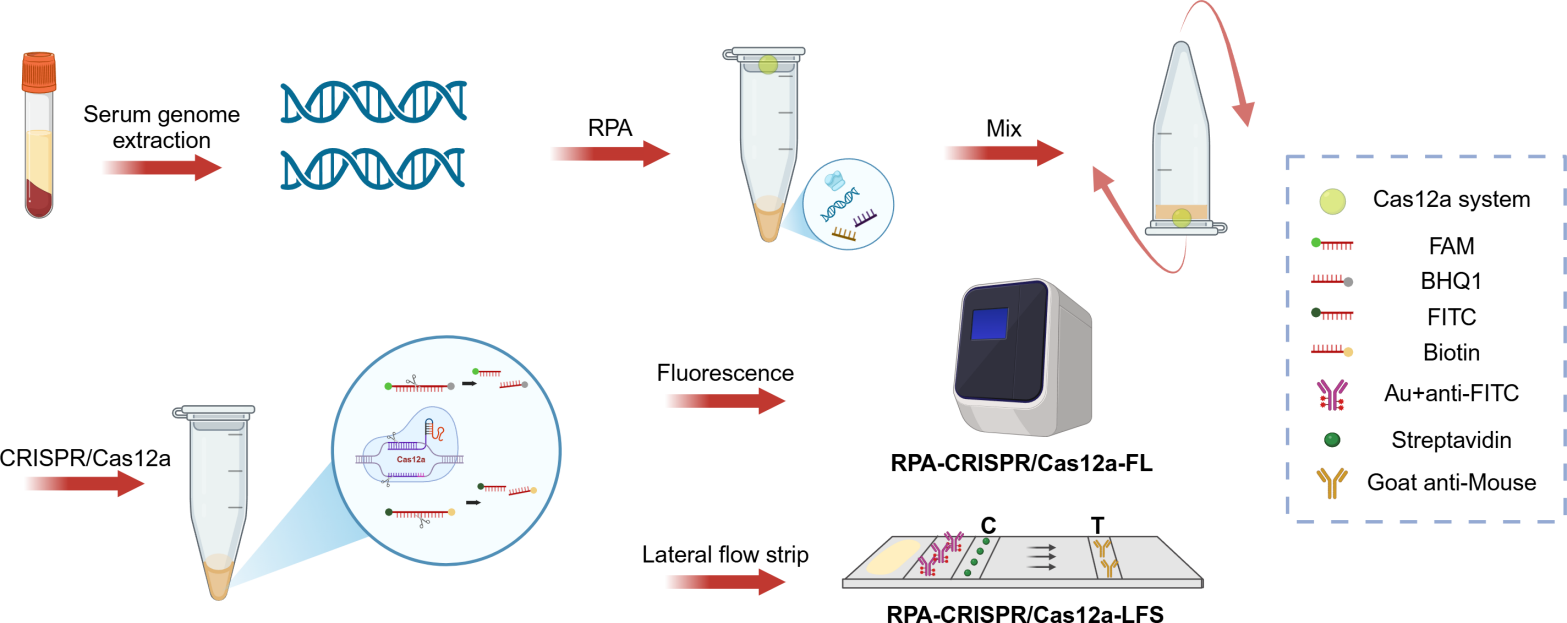
The diagram of *B. melitensis* detection based on RPA-CRISPR/Cas12a-FL and RPA-CRISPR/Cas12a-LFS. Genomic DNA extracted from the serum is amplified by the RPA. In the case of a positive sample, crRNA binds to Cas12a to specifically identify the target gene. Subsequently, Cas12a can nonspecifically cut FAM-BHQ1 and FITC-biotin reporter genes. The separation of FAM from BHQ1 enables FL detection by qPCR instrument. In addition, the separation of FITC from biotin makes the Au-nanoparticle anti-FITC bound to it unable to be fully captured by streptavidin in the quality control line, thus forming a visible band on the detection line.

**Fig 2:**
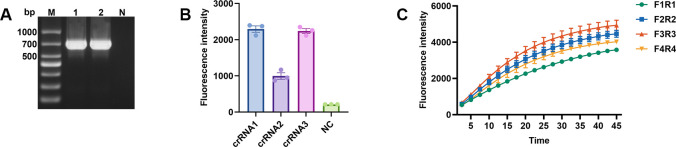
The screening of the RPA-CRISPR/Cas12a assay crRNA and primers. (**A**) PCR amplification of the *B. melitensis* omp31 gene. M, DNA marker; 1–2, omp31 gene; and N, negative control. (**B**) Comparison of different crRNAs targeting omp31 by FL assay, N, negative control. (**C**) Screening results for RPA primers.

**Fig 3:**
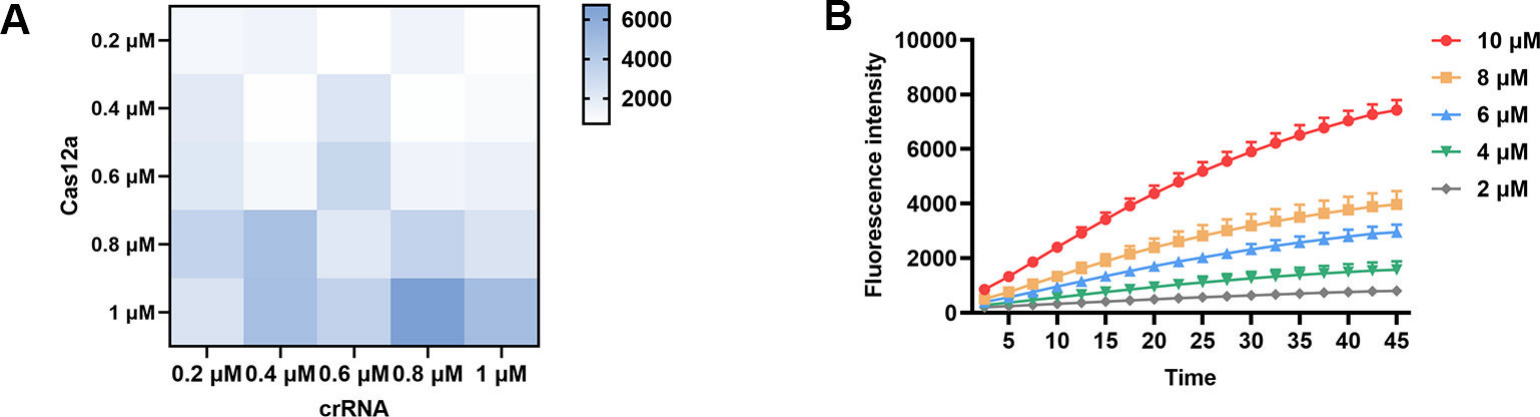
Optimization results of the RPA-CRISPR/Cas12a assay. (**A**) Optimization of the concentration of Cas12a and crRNA. (**B**) Optimization of the concentration of reporter.

In this study, we developed a rapid detection method for *Brucella* infections that combines RPA with the CRISPR/Cas12a system, and the final results can be presented by fluorescent or lateral flow strip (LFS), as shown in Fig. 1. In order to improve the detection sensitivity, crRNA screening, primer screening, and optimization of experimental conditions were systematically carried out in our study. Firstly, the whole genome of *Brucella* was used as a template, and the omp31 gene (Fig. 2A) was amplified by PCR using primers omp31-F and omp31-R (Table S1), and the plasmid PMD-19T-omp31 was obtained by cloning the gene into the pMD-19T vector. Subsequently, for the omp31 gene, three sets of crRNAs were designed in this study (Table S1), and their performance was compared by evaluating the FL intensity of the reaction. After comparison, crRNA1 was determined to be the best choice for brucellosis detection (Fig. 2B). Regarding RPA amplification primer screening, four pairs of primers were designed and evaluated in this study (Table S1), and the optimal primer combination, F3R3, was finally determined (Fig. 2C). By optimizing the concentrations of Cas12a protein and crRNA through the checkerboard method, we finally determined that the best detection results were achieved at a protein concentration of 1 µM and a crRNA concentration of 0.8 µM (Fig. 3A). In addition, the optimal concentration of the probe was also explored in this study, and after a series of dilution experiments, the probe concentration was finally determined to be 10 µM (Fig. 3B).

### RPA-CRISPR/Cas12a assay has robust sensitivity

**Fig 4:**
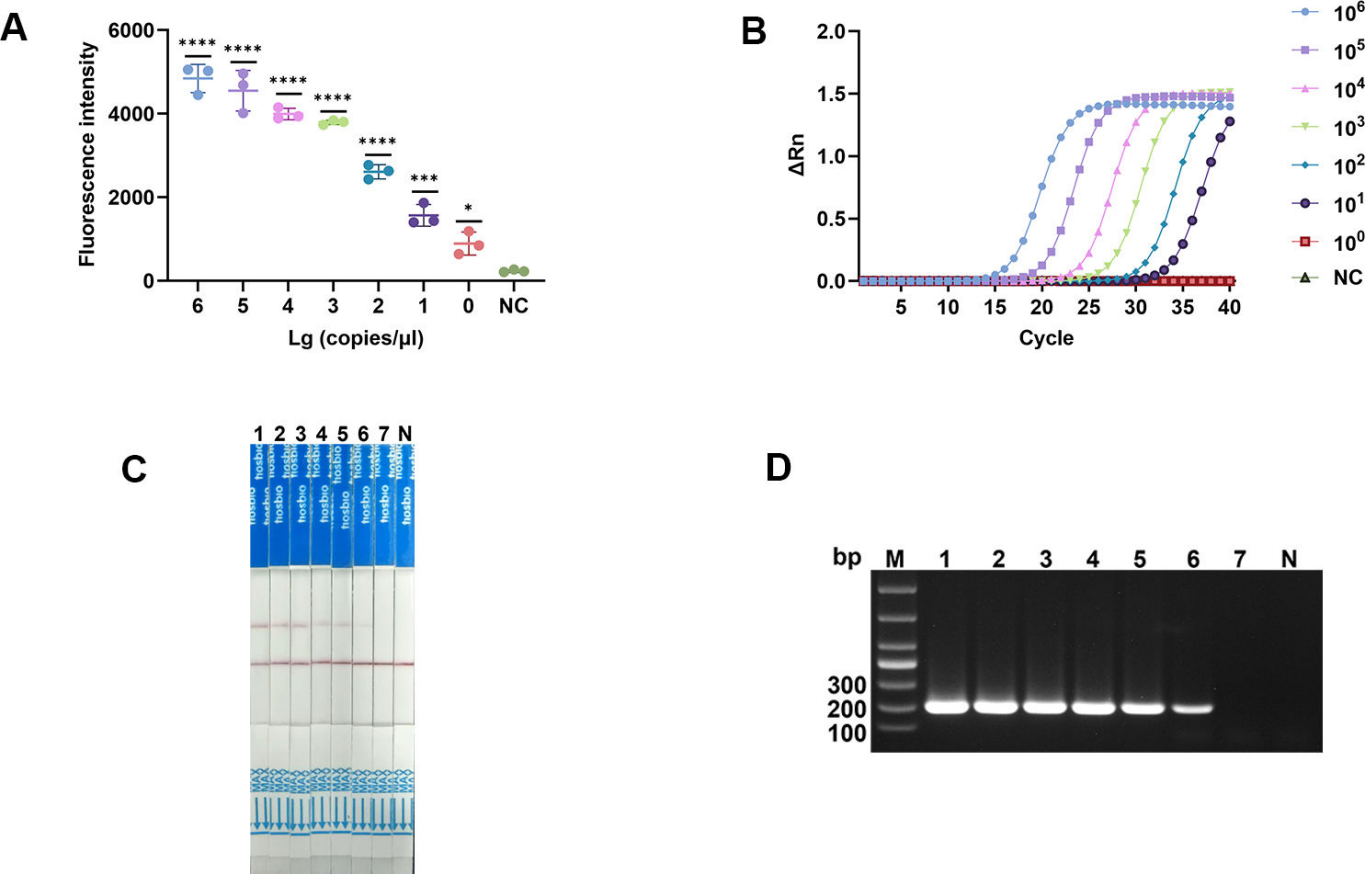
The sensitivity analysis of RPA-CRISPR/Cas12a-FL and RPA-CRISPR/Cas12a-LFS. (**A**) Sensitivity result of the RPA-CRISPR/Cas12a-FL assay was performed using serial 10-fold dilutions of pMD-19T-omp31 as a template. The concentration gradients detected were 1 × 10^6^ copies/μL, 1 × 10^5^ copies/μL, 1 × 10^4^ copies/μL, 1 × 10^3^ copies/μL, 1 × 10^2^ copies/μL, 1 × 10^1^ copies/μL, and 1 × 10^0^ copy/μL, corresponding to numbers 6 to 0, respectively. NC, negative control. (**B**) Sensitivity result of the qPCR. NC, negative control. (**C and D**) Sensitivity result of the RPA-CRISPR/Cas12a-LFS assay and nested PCR was performed using serial 10-fold dilutions of pMD-19T-omp31 as a template. The concentration gradients detected were 1 × 10^6^ copies/μL, 1 × 10^5^ copies/μL, 1 × 10^4^ copies/μL, 1 × 10^3^ copies/μL, 1 × 10^2^ copies/μL, 1 × 10^1^ copies/μL, and 1 × 10^0^ copy/μL, corresponding to numbers 1 to 7, respectively. N, negative control. ****P < 0.0001; ***P < 0.001; **P < 0.01; *P < 0.1.

We used different concentrations of pMD-19T-omp31 plasmid for RPA-CRISPR/Cas12a sensitivity analysis. Statistical analysis through an independent student’s t-test showed that the FL intensity of plasmids with concentrations ranging from 1 × 10^6^ to 1 × 10^1^ copies/μL was significantly higher than that of the negative control group, and the FL intensity of plasmids with a concentration of 1 × 10^0^ copy/μL was also higher than that of the negative control group. The result showed that the lower limit of detection (LOD) of the RPA-CRISPR/Cas12a-FL method was 1 copy/μL (Fig. 4A). In addition, through LFS detection, we can visually observe that the LOD of the RPA-CRISPR/Cas12a-LFS method was 10 copies/μL (Fig. 4B). Meanwhile, we also performed fluorescent PCR and nested PCR assays (Fig. 4C and D). The results showed that the sensitivity of the RPA-CRISPR/Cas12a-FL assay was 10 times higher than that of qPCR, and the sensitivity of the RPA-CRISPR/Cas12a-LFS assay was comparable to that of nested PCR.

### RPA-CRISPR/Cas12a assay has robust specificity

**Fig 5:**
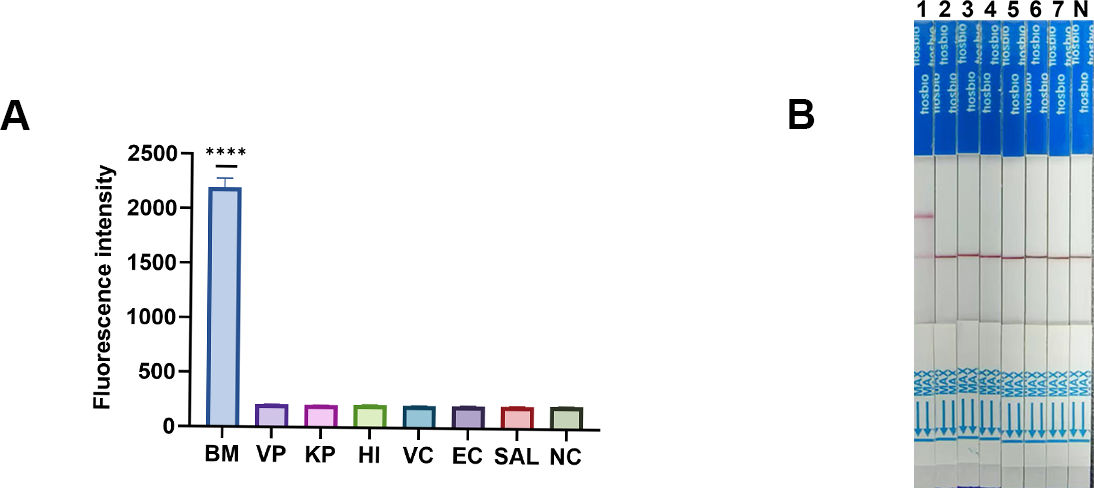
The specificity analysis of RPA-CRISPR/Cas12a-FL and RPA-CRISPR/Cas12a-LFS. (**A**) Sensitivity result of the RPA-CRISPR/Cas12a-FL assay was performed using DNA from different bacterial species. (**B**) Sensitivity result of the nested PCR was performed using DNA from different bacterial species. 1–7 represent BM, VP, KP, HI, VC, EC, and SAL. N, negative control. ****P < 0.0001.

To analyze the specificity of the RPA-CRISPR/Cas12a assay, we used DNA samples from a variety of different bacteria, including *Vibrio paraholyticus*, *Klebsiella pneumoniae*, *Haemophilus influenzae*, *V. cholerae*, *Escherichia coli,* and *Salmonella*. As shown in Fig. 5A, the RPA-CRISPR-Cas12a-FL FL assay showed that only *Brucella* showed a strong fluorescent signal, whereas none of the other bacteria and negative control samples showed a signal. In addition, the RPA-CRISPR-Cas12a-LFS lateral flow immunoassay showed that the *Brucella* samples had a clear positive color band, while the other bacterial species did not show any positive color band (Fig. 5B). These results indicate that both methods have excellent detection specificity.

### RPA-CRISPR/Cas12a assay detection for serum sample

To validate the feasibility of this assay for clinical application, serum samples were collected from 24 confirmed patients with brucellosis and six healthy controls. All samples were serologically validated. After extracting the sample genomes, fluorescent PCR and RPA-CRISPR/Cas12a assays were performed, and the results are shown in Table 1. The two assays were consistent with the serologic results, indicating the clinical application of the method.

**TABLE 1:** Serum samples test

Detection method	Serum samples (*n* = 30)	
	Positive	Negative
RPA-CRISPR/Cas12a-FL	24	6
RPA-CRISPR/Cas12a-LFS	24	6
qPCR	24	6

## DISCUSSION

The development of a rapid and sensitive diagnostic assay for detecting *B. melitensis* is critical in both clinical and veterinary settings, given the significant public health and economic implications of brucellosis. This study successfully developed a novel detection method based on RPA-CRISPR/Cas12a, which combines the rapidity of RPA with the high specificity of CRISPR/Cas12a technology. This assay provides a promising alternative to traditional methods such as culture-based techniques, serological assays, and conventional PCR.

Traditional diagnostic methods for *B. melitensis* exhibit several limitations. Culture-based methods, while considered the gold standard are time-consuming, requiring several days to weeks for bacterial growth and identification (11, 29). Additionally, they pose a significant risk to laboratory personnel due to the highly infectious nature of *Brucella* species (30, 31). On the other hand, serological assays are faster but often suffer from cross-reactivity with other pathogens, leading to false-positive results (32). Conventional PCR and real-time PCR offer improved sensitivity and specificity but require sophisticated equipment and skilled personnel, limiting their use in resource-limited settings (33, 34).

In contrast, our detection method based on RPA-CRISPR/Cas12a effectively addresses many of the limitations mentioned above. The RPA technology can rapidly amplify the target DNA at a constant temperature (37–42°C) without the need for thermal cycling, thereby shortening the amplification time to within 20 min (19, 35). By integrating CRISPR/Cas12a technology, the target sequence can be specifically recognized, and after recognition, it exhibits significant trans-cleavage activity, which cleaves the single-stranded fluorescent reporter molecule, generating a detectable signal (36, 37). This combination of RPA and CRISPR/Cas12a results in a detection method characterized by high sensitivity and strong specificity. The entire detection process can be completed within 1 h, making it particularly suitable for rapid and accurate clinical diagnosis (38, 39).

This study evaluates detection methods based on FL and LFSs, each possessing unique advantages in a diagnostic setting. The sensitivity of the RPA-CRISPR/Cas12a assay was rigorously evaluated using various concentrations of the pMD-19T-omp31 plasmid. The FL-based method (RPA-CRISPR/Cas12a-FL) exhibited a LOD of 1 copy/μL, whereas the LFS method (RPA-CRISPR/Cas12a-LFS) demonstrated an LOD of 10 copies/μL. As shown in Fig. 4B, this sensitivity level is significantly higher than that of conventional qPCR. The enhanced sensitivity of the RPA-CRISPR/Cas12a assay can be attributed to the synergistic combination of RPA, which facilitates rapid and efficient amplification of target DNA at a constant temperature, and the CRISPR/Cas12a system, which offers a highly specific and sensitive detection mechanism through its trans-cleavage activity (24, 40). The specificity of the assay was confirmed by testing against a panel of DNA samples from various bacterial species. The results indicated that only *Brucella* samples produced a strong fluorescent signal or a positive color band, while no signal was observed for the other bacterial species or negative controls. This high specificity aligns with previous studies utilizing CRISPR-based systems for pathogen detection, underscoring the capability of CRISPR/Cas12a to distinguish target sequences with single-nucleotide precision (41, 42). The absence of cross-reactivity with other pathogens highlights the robustness of the assay and its potential for accurate diagnosis in both clinical and field settings.

The clinical applicability of the RPA-CRISPR/Cas12a assay was validated using serum samples from 24 confirmed brucellosis patients and six healthy controls. The assay results were consistent with serological findings, demonstrating its potential for use in clinical diagnostics. The ability to detect *Brucella* DNA in serum samples is particularly important, as serum is a commonly used sample type for brucellosis diagnosis (43).

Despite the promising results, this study has several limitations. First, the assay was primarily validated using laboratory-prepared plasmid DNA and a limited number of clinical serum samples. Further validation with a larger cohort of clinical samples, including those from various stages of infection and different geographical regions, is essential to confirm its robustness and reliability in diverse settings. Second, the assay’s performance in detecting other *Brucella* species, such as *B. abortus* and *B. suis*, has yet to be evaluated. Developing a detection method for multiple *Brucella* species simultaneously can not only improve the detection sensitivity of different *Brucella* species but also enable rapid diagnosis of multiple *Brucella* species in epidemic areas, which is conducive to molecular epidemiological investigations and pathogen traceability. Third, while the LFS format offers simplicity and portability, its sensitivity is slightly lower than that of the FL-based method. Future research could focus on optimizing the LFS to improve its sensitivity without compromising ease of use. For example, the reaction time can be appropriately extended, the replacement of other manufacturers of nucleic acid test strips or combined with digital visualization instruments to read the results.

### Conclusion

In conclusion, the RPA-CRISPR/Cas12a-based assay developed in this study offers a rapid, sensitive, and specific method for the detection of *B. melitensis*. The simplicity, speed, and point-of-care testing potential of the assay make it a valuable addition to the existing diagnostics for brucellosis. By addressing the limitations of traditional and molecular methods, the RPA-CRISPR/Cas12a assay has the potential to significantly improve the diagnosis and control of *B. melitensis* infections, ultimately reducing the burden of brucellosis on public health and agriculture.

## MATERIALS AND METHODS

### Samples collection and DNA extraction

The *B. melitensis*, *V. paraholyticus*, *K. pneumoniae*, *H. influenzae*, *V. cholerae*, *E. coli,* and *Salmonella* were stored in our laboratory. All strains were preserved at −70°C using Microbank preservation vials (Pro-Lab Diagnostics, Ontario, Canada) according to the manufacturer’s protocol. A total of 30 serum samples were collected from various hospitals in Hangzhou, Zhejiang Province. Bacterial and serum genomic DNA was extracted using the DNeasy Blood & Tissue Kit (QIAGEN, Hilden, Germany) according to the manufacturer’s protocol and stored at −20°C for subsequent use.

### Construction of the positive plasmid

The omp31 gene fragment was expanded by PCR reaction using *Brucella* genome extracted above as a template. The PCR was carried out in a volume of 50 µL containing 25 µL of 2 × HotStart Taq PCR Mix (TIANGEN, Beijing, China), 2 µL of each primer (10 µmol/L), 1 µL genomic DNA template, and 20 µL of sterile distilled water by the conditions with an initial melting step at 94°C for 3 min, followed by 30 cycles with each cycle at 94°C for 30 s, 60°C for 30 s, and 72°C for 1 min, followed by a final extension at 72°C for 5 min. Subsequently, we added the A base to the 3′ end of the PCR product using a DNA A-tail Kit (Takara, Kusatsu, Japan). The DNA fragment with the A-tail was then cloned into the pMD19-T vector (Takara). After transformation and resistance plate inoculation, positive clones were screened and sequenced. Finally, the recombinant plasmid pMD-19T-omp31 was obtained and stored at −20°C for subsequent use.

### crRNA design and screen

Three crRNAs were designed using conserved and specific *Brucella* omp31 gene (GenBank: GQ184729) as target genes. The specific sequences of crRNA and single-strand probes (ssDNA) were shown in Table S1, which were synthesized by the Sangon Biotech Co., Ltd. (Shanghai, China). In order to screen out the optimal crRNA, we performed a CRISPR/Cas12a assay. In addition, three independent replicates were performed for each set of experiments. The assay system consisted of 1 µL Cas12a protein (New England Biolabs, MA, USA), 1 µL crRNA, 3 µL 10 × NEBuffer 2.0 (New England Biolabs), 1 µL ssDNA probe, 2.5 µL pMD-19T-omp31 gene, and 21.5 µL of sterile distilled water. The reaction tube was placed in the LineGene 9600 Plus instrument (Bioer Tech, Zhejiang, China), and the FL signal was detected at 37°C for 45 min. FL intensity was recorded and used to evaluate the cleavage efficiency of crRNA.

### Establishment of RPA-CRISPR/Cas12a one-tube assay

RPA primers were designed with Primer Premier six software (Premier Biosoft, San Francisco, CA, USA) according to the primer design guidelines of the TwistDx company and synthesized by the Sangon Biotech Co., Ltd. Subsequently, the optimal crRNA obtained from the above screening was used to react with these primers, respectively, and finally, the optimal RPA primer was found. The specific reactions were divided into RPA and CRISPR/Cas12a systems. The TwistAmp Basic Kit (TwistDX Cambridge, UK) was used for RPA amplification in a system consisting of 1.2 µL each RPA primer (10 µM), 14.5 µL buffer, 2.5 µL plasmid template, and 1.2 µL MgOAc (280 mM). The CRISPR/Cas12a system consists of 1 µL Cas12a protein, 1 µL crRNA, 1 µL ssDNA probe, and 3 µL 10× NEBuffer 2.0. The RPA system was first added to the PCR tube, and then the CRISPR/Cas12a system was placed on the cap of the PCR tube. After the RPA reaction for 15 min, the two systems were mixed and reacted at 37℃ in a fluorescent PCR instrument for 45 min. Each group of experiments was independently repeated three times. The final FL intensity was recorded to evaluate the optimal RPA primer pair and the feasibility of the reaction.

### Optimization of RPA-CRISPR/Cas12a reaction conditions

In single-tube RPA-CRISPR/Cas12a assays, the specific concentration of Cas12a protein and crRNA is critical to obtain the best detection results. In this study, the chessboard method was used to determine the optimal Cas12a and crRNA concentrations (1 µM, 0.8 µM, 0.6 µM, 0.4 µM, and 0.2 µM) by analyzing the FL intensity. In addition, this study also optimized the concentration of the single-strand probe on this basis.

### RPA-CRISPR/Cas12a combined with LFS

Based on the above RPA FL detection method, this study also established a lateral flow test strip detection. For the LFS detection, the system is the same as the FL detection method; only the probe is changed to biotin labeling, and the Cas reaction time is adjusted to 25 min. After the reaction, 70 µL PBS buffer was added into the test tube, thoroughly mixed, and vertically inserted into the LFS (Tiosbio, Beijing, China). The results were interpreted within 5–10 min.

### Sensitivity and specificity analysis of RPA-CRISPR/Cas12a assay

To determine the detection limit of RPA-CRISPR/Cas12a, the plasmid containing pMD-19T-omp31 was diluted to 1 × 10^6^ copies/μL, 1 × 10^5^ copies/μL, 1 × 10^4^ copies/μL, 1 × 10^3^ copies/μL, 1 × 10^2^ copies/μL, 1 × 10^1^ copies/μL, and 1 × 10^0^ copy/μL, respectively. The sensitivity of RPA-CRISPR-Cas12a FL detection and RPA-CRISPR-Cas12a LFS detection was tested, respectively. The experimental results were compared with those of qPCR and nested PCR. The qPCR was carried out in a volume of 50 µL containing 25 µL of 2× Real-time PCR Master Mix (Toyobo, Osaka, Japan), 1 µL of each primer (Table S1), 2.5 µL of DNA template, and 20.5 µL of sterile distilled water, and amplification was performed on a LineGene 9600 Plus instrument (Bioer Tech). The nested PCR was carried out in a volume of 50 µL containing 25 µL of 2 × HotStart Taq PCR Mix (TIANGEN), 2 µL of each primer (10 µmol/L), 2.5 µL of genomic DNA template, and 18.5 µL of sterile distilled water by the conditions with an initial melting step at 94°C for 3 min, followed by 30 cycles with each cycle at 94°C for 30 s, 60°C for 30 s, and 72°C for 1 min, followed by a final extension at 72°C for 5 min. 1 µL of the product of the first round is added to the system of the second round as the template of the second round, and the procedure is the same as the first round. To verify the accuracy of the results, three detections were performed for each concentration of pMD-19T-omp31. In addition, to assess specificity, both methods tested DNA from six non-target bacteria, including *V. paraholyticus*, *K. pneumoniae*, *H. influenzae*, *V. cholerae*, *E. coli,* and *Salmonella*.

### Clinical sample testing

The RPA-CRISPR/Cas12a and RPA-CRISPR/ Cas12A-LFS detection methods established in this study were used to detect *Brucella* in 30 serologically negative or positive samples collected from different hospitals in Hangzhou, Zhejiang Province.

## SUPPLEMENTAL MATERIAL

Table S1 spectrum.00998-25-s0001.pdf Primer sequence.

ASM does not own the copyrights to Supplemental Material that may be linked to, or accessed through, an article. The authors have granted ASM a non-exclusive, world-wide license to publish the Supplemental Material files. Please contact the corresponding author directly for reuse.

## References

[1] Freire ML, Machado de Assis TS, Silva SN, Cota G. 2024. Diagnosis of human brucellosis: systematic review and meta-analysis. PLoS Negl Trop Dis 18:e0012030. doi:10.1371/journal.pntd.0012030 38452046 PMC10950246

[2] Kaynak-Onurdag F, Okten S, Sen B. 2016. Screening Brucella spp. in bovine raw milk by real-time quantitative PCR and conventional methods in a pilot region of vaccination, Edirne, Turkey. J Dairy Sci 99:3351–3357. doi:10.3168/jds.2015-10637 26971148

[3] Franco MP, Mulder M, Gilman RH, Smits HL. 2007. Human brucellosis. Lancet Infect Dis 7:775–786. doi:10.1016/S1473-3099(07)70286-4 18045560

[4] Franc KA, Krecek RC, Häsler BN, Arenas-Gamboa AM. 2018. Brucellosis remains a neglected disease in the developing world: a call for interdisciplinary action. BMC Public Health 18:125. doi:10.1186/s12889-017-5016-y 29325516 PMC5765637

[5] Dadar M, Shahali Y, Whatmore AM. 2019. Human brucellosis caused by raw dairy products: a review on the occurrence, major risk factors and prevention. Int J Food Microbiol 292:39–47. doi:10.1016/j.ijfoodmicro.2018.12.009 30572264

[6] Oliver SP, Boor KJ, Murphy SC, Murinda SE. 2009. Food safety hazards associated with consumption of raw milk. Foodborne Pathog Dis 6:793–806. doi:10.1089/fpd.2009.0302 19737059

[7] Laine CG, Johnson VE, Scott HM, Arenas-Gamboa AM. 2023. Global estimate of human brucellosis incidence. Emerg Infect Dis 29:1789–1797. doi:10.3201/eid2909.230052 37610167 PMC10461652

[8] Mantur BG, Amarnath SK, Shinde RS. 2007. Review of clinical and laboratory features of human brucellosis. Indian J Med Microbiol 25:188–202. doi:10.4103/0255-0857.34758 17901634

[9] Zheng R, Xie S, Lu X, Sun L, Zhou Y, Zhang Y, Wang K. 2018. A systematic review and meta-analysis of epidemiology and clinical manifestations of human brucellosis in China. Biomed Res Int 2018:5712920. doi:10.1155/2018/5712920 29850535 PMC5937618

[10] Pappas G, Akritidis N, Bosilkovski M, Tsianos E. 2005. Brucellosis. N Engl J Med 352:2325–2336. doi:10.1056/NEJMra050570 15930423

[11] Yagupsky P, Morata P, Colmenero JD. 2019. Laboratory diagnosis of human brucellosis. Clin Microbiol Rev 33:e00073-19. doi:10.1128/CMR.00073-19 31722888 PMC6860005

[12] Di Bonaventura G, Angeletti S, Ianni A, Petitti T, Gherardi G. 2021. Microbiological laboratory diagnosis of human brucellosis: an overview. Pathogens 10:1623. doi:10.3390/pathogens10121623 34959578 PMC8709366

[13] Memish Z, Mah MW, Al Mahmoud S, Al Shaalan M, Khan MY. 2000. Brucella bacteraemia: clinical and laboratory observations in 160 patients. J Infect 40:59–63. doi:10.1053/jinf.1999.0586 10762113

[14] Araj GF. 2010. Update on laboratory diagnosis of human brucellosis. Int J Antimicrob Agents 36 Suppl 1:S12–7. doi:10.1016/j.ijantimicag.2010.06.014 20692128

[15] Dang S, Sui H, Zhang S, Wu D, Chen Z, Zhai J, Bai M. 2023. CRISPR-Cas12a test strip (CRISPR/CAST) package: in-situ detection of Brucella from infected livestock. BMC Vet Res 19:202. doi:10.1186/s12917-023-03767-1 37833763 PMC10571365

[16] Mitka S, Anetakis C, Souliou E, Diza E, Kansouzidou A. 2007. Evaluation of different PCR assays for early detection of acute and relapsing brucellosis in humans in comparison with conventional methods. J Clin Microbiol 45:1211–1218. doi:10.1128/JCM.00010-06 17267626 PMC1865811

[17] Becker GN, Tuon FF. 2021. Comparative study of IS711 and bcsp31-based polymerase chain reaction (PCR) for the diagnosis of human brucellosis in whole blood and serum samples. J Microbiol Methods 183:106182. doi:10.1016/j.mimet.2021.106182 33647359

[18] Kaden R, Ferrari S, Alm E, Wahab T. 2017. A novel real-time PCR assay for specific detection of Brucella melitensis. BMC Infect Dis 17:230. doi:10.1186/s12879-017-2327-7 28340558 PMC5366107

[19] Piepenburg O, Williams CH, Stemple DL, Armes NA. 2006. DNA detection using recombination proteins. PLoS Biol 4:e204. doi:10.1371/journal.pbio.0040204 16756388 PMC1475771

[20] Asa TA, Kumar P, Lee J, Seo YJ. 2023. Multiple ligation-assisted recombinase polymerase amplification for highly sensitive and selective colorimetric detection of SARS-CoV-2. Talanta 252:123835. doi:10.1016/j.talanta.2022.123835 35985194 PMC9375730

[21] Qin L, Nan W, Wang Y, Zhang Y, Tan P, Chen Y, Mao K, Chen Y. 2019. A novel approach for detection of brucella using a real-time recombinase polymerase amplification assay. Mol Cell Probes 48:101451. doi:10.1016/j.mcp.2019.101451 31541671

[22] Gumaa MM, Cao X, Li Z, Lou Z, Zhang N, Zhang Z, Zhou J, Fu B. 2019. Establishment of a recombinase polymerase amplification (RPA) assay for the detection of Brucella spp. Infection. Mol Cell Probes 47:101434. doi:10.1016/j.mcp.2019.101434 31401295 PMC7127669

[23] Zheng T, Li X, Si Y, Wang M, Zhou Y, Yang Y, Liang N, Ying B, Wu P. 2023. Specific lateral flow detection of isothermal nucleic acid amplicons for accurate point-of-care testing. Biosens Bioelectron 222:114989. doi:10.1016/j.bios.2022.114989 36538868

[24] Zetsche B, Gootenberg JS, Abudayyeh OO, Slaymaker IM, Makarova KS, Essletzbichler P, Volz SE, Joung J, van der Oost J, Regev A, Koonin EV, Zhang F. 2015. Cpf1 is a single RNA-guided endonuclease of a class 2 CRISPR-Cas system. Cell 163:759–771. doi:10.1016/j.cell.2015.09.038 26422227 PMC4638220

[25] Li X, Zhu S, Zhang X, Ren Y, He J, Zhou J, Yin L, Wang G, Zhong T, Wang L, Xiao Y, Zhu C, Yin C, Yu X. 2023. Advances in the application of recombinase-aided amplification combined with CRISPR-Cas technology in quick detection of pathogenic microbes. Front Bioeng Biotechnol 11:1215466. doi:10.3389/fbioe.2023.1215466 37720320 PMC10502170

[26] Broughton JP, Deng X, Yu G, Fasching CL, Servellita V, Singh J, Miao X, Streithorst JA, Granados A, Sotomayor-Gonzalez A, Zorn K, Gopez A, Hsu E, Gu W, Miller S, Pan C-Y, Guevara H, Wadford DA, Chen JS, Chiu CY. 2020. CRISPR-Cas12-based detection of SARS-CoV-2. Nat Biotechnol 38:870–874. doi:10.1038/s41587-020-0513-4 32300245 PMC9107629

[27] Compiro P, Chomta N, Nimnual J, Sunantawanit S, Payungporn S, Rotcheewaphan S, Keawsapsak P. 2025. CRISPR-Cas12a-based detection and differentiation of Mycobacterium spp. Clin Chim Acta 567:120101. doi:10.1016/j.cca.2024.120101 39725131

[28] Wei H, Li J, Liu Y, Cheng W, Huang H, Liang X, Huang W, Lin L, Zheng Y, Chen W, Wang C, Chen W, Xu G, Wei W, Chen L, Zeng Y, Lu Z, Li S, Lin Z, Wang J, Lin M. 2023. Rapid and ultrasensitive detection of Plasmodium spp. parasites via the RPA-CRISPR/Cas12a platform. ACS Infect Dis 9:1534–1545. doi:10.1021/acsinfecdis.3c00087 37493514

[29] Pappas G, Papadimitriou P, Akritidis N, Christou L, Tsianos EV. 2006. The new global map of human brucellosis. Lancet Infect Dis 6:91–99. doi:10.1016/S1473-3099(06)70382-6 16439329

[30] Al Dahouk S, Nöckler K. 2011. Implications of laboratory diagnosis on brucellosis therapy. Expert Rev Anti Infect Ther 9:833–845. doi:10.1586/eri.11.55 21810055

[31] Yagupsky P, Baron EJ. 2005. Laboratory exposures to brucellae and implications for bioterrorism. Emerg Infect Dis 11:1180–1185. doi:10.3201/eid1108.041197 16102304 PMC3320509

[32] Díaz R, Casanova A, Ariza J, Moriyón I. 2011. The rose bengal test in human brucellosis: a neglected test for the diagnosis of a neglected disease. PLoS Negl Trop Dis 5:e950. doi:10.1371/journal.pntd.0000950 21526218 PMC3079581

[33] Yu WL, Nielsen K. 2010. Review of detection of Brucella spp. by polymerase chain reaction. Croat Med J 51:306–313. doi:10.3325/cmj.2010.51.306 20718083 PMC2931435

[34] Craw P, Balachandran W. 2012. Isothermal nucleic acid amplification technologies for point-of-care diagnostics: a critical review. Lab Chip 12:2469–2486. doi:10.1039/c2lc40100b 22592150

[35] Lobato IM, O’Sullivan CK. 2018. Recombinase polymerase amplification: basics, applications and recent advances. Trends Analyt Chem 98:19–35. doi:10.1016/j.trac.2017.10.015 PMC7112910 32287544

[36] Paul B, Montoya G. 2020. CRISPR-Cas12a: functional overview and applications. Biomed J 43:8–17. doi:10.1016/j.bj.2019.10.005 32200959 PMC7090318

[37] Mao Z, Chen R, Wang X, Zhou Z, Peng Y, Li S, Han D, Li S, Wang Y, Han T, Liang J, Ren S, Gao Z. 2022. CRISPR/Cas12a-based technology: a powerful tool for biosensing in food safety. Trends Food Sci Technol 122:211–222. doi:10.1016/j.tifs.2022.02.030 35250172 PMC8885088

[38] Sun Y, Yu L, Liu C, Ye S, Chen W, Li D, Huang W. 2021. One-tube SARS-CoV-2 detection platform based on RT-RPA and CRISPR/Cas12a. J Transl Med 19:74. doi:10.1186/s12967-021-02741-5 33593370 PMC7884969

[39] Liu Y, Chao Z, Ding W, Fang T, Gu X, Xue M, Wang W, Han R, Sun W. 2024. A multiplex RPA-CRISPR/Cas12a-based POCT technique and its application in human papillomavirus (HPV) typing assay. Cell Mol Biol Lett 29:34. doi:10.1186/s11658-024-00548-y 38459454 PMC10921630

[40] Fonfara I, Richter H, Bratovič M, Le Rhun A, Charpentier E. 2016. The CRISPR-associated DNA-cleaving enzyme Cpf1 also processes precursor CRISPR RNA. Nature New Biol 532:517–521. doi:10.1038/nature17945 27096362

[41] Swarts DC, Jinek M. 2019. Mechanistic insights into the cis- and trans-acting DNase activities of Cas12a. Mol Cell 73:589–600. doi:10.1016/j.molcel.2018.11.021 30639240 PMC6858279

[42] Swarts DC. 2019. Making the cut(s): how Cas12a cleaves target and non-target DNA. Biochem Soc Trans 47:1499–1510. doi:10.1042/BST20190564 31671185

[43] Al Dahouk S, Tomaso H, Nöckler K, Neubauer H, Frangoulidis D. 2003. Laboratory-based diagnosis of brucellosis--a review of the literature. Part II: serological tests for brucellosis. Clin Lab 49:577–589. 14651329

