# Supplementary material for: Development of a rapid and sensitive RPA-CRISPR/Cas12a-based assay for the detection of Brucella melitensis
Source: Microbiol Spectr. 2025 Aug 27;13(10):e00998-25. doi: 10.1128/spectrum.00998-25 (PMC12502682; doi:10.1128/spectrum.00998-25)
Supplement: Table S1 — Primer sequence. [file spectrum.00998-25-s0001.pdf]

Supplementary information

Supplementary Table1. The primers used in this study.

Primer	Sequence (5'to3')	Use
OMP31-F	ATGAAATCCGTAATTTTGGCGTCCATCG	Amplification omp31
OMP31-R	TTAGAACTTGTAGTTCAGACCGACGCGA	Amplification omp31
crRNA1	UAAUUUCUACUAAGUGUAGAU<br>GUCUUGUCGGACCACGUGUG	CRISPR/Cas12a
crRNA2	UAAUUUCUACUAAGUGUAGAU<br>UGAACCUUCCGCCCCCACUGCU	
crRNA3	UAAUUUCUACUAAGUGUAGAU<br>UGGUACCGGCGGUCUGGCCUAU	
F1	CTGGCCTATGGTAAGGTCAAGTCTGCGTTC	RPA
R1	TTGAGCGTCCAGTTGTTGTTGATGGCATATTC	
F2	GCGGTCTGGCCTATGGTAAGGTCAAGTCTG	
R2	CTTGAGCGTCCAGTTGTTGTTGATGGCATATTC	
F3	TACACGGCTACCGAACGCCTCATGGTTTATG	
R3	TATTCCGACTTGAGCGTCCAGTTGTTGTTGATG	
F4	TCAAGTCTGCGTTCAACCTGGGTGATGATG	
R4	TGCTCTCAAGGAAGCTATTGTCAACGTCGA	
ssDNA1	FAM-TTATT-BHQ1	Fluorescence detection
ssDNA2	FITC-TTATT- Biotin	Lateral flow test strip detection
qOMP31-F	CTGGACGCTCAAGTCGGAAT	omp31-based qPCR
qOMP31-R	TCTCAAGGAAGCTATTGTCAACGT	
OMP31-N-F1	AGGCGGCAAGTTCAAGCATCC	omp31 outer primer for nested PCR
OMP31-N-R1	AACCATGAGGCGTTCGGTAGC	
OMP31-N-F2	GGCTTCGTCGGTGGTGTTCA	omp31 inner primer for nested PCR
OMP31-N-R2	TCGGTAGCCGTGTAGCCAAGA	
